# Detection of Cellular Senescence Reveals the Existence of Senescent Tumor Cells within Invasive Breast Carcinomas and Related Metastases

**DOI:** 10.3390/cancers15061860

**Published:** 2023-03-20

**Authors:** Cristina L. Cotarelo, Arno Schad, Marcus Schmidt, Arnd Hönig, Jonathan P. Sleeman, Sonja Thaler

**Affiliations:** 1 Institute of Pathology, University Medical Center Mannheim, University of Heidelberg, 68167 Mannheim, Germany; 2 Institute of Pathology, University Medical Center, Johannes Gutenberg University, 55131 Mainz, Germany; 3 Department of Gynecology and Obstetrics, University Medical Center, Johannes Gutenberg University, 55131 Mainz, Germany; 4 Breast Center, Women’s Hospital, Marienhaus Hospital Mainz, 55131 Mainz, Germany; 5 European Center for Angioscience, Medical Faculty Mannheim, University of Heidelberg, 68167 Mannheim, Germany; 6 Institute of Biological and Chemical Systems—Biological Information Processing (IBCS-BIP), Karlsruhe Institute of Technology (KIT), Campus Nord, 76344 Eggenstein-Leopoldshafen, Germany

**Keywords:** cellular senescence, metastatic breast cancer, senescent breast cancer cells

## Abstract

**Simple Summary:**

Oncogene-induced senescence constitutes a barrier to carcinogenesis by arresting cells at risk of malignant transformation. However, numerous findings suggest that senescent cells conversely promote tumor growth and metastatic progression, for example, through the senescence-associated secretory phenotype (SASP) they produce. In patients with luminal A and B breast carcinomas, we found broad similarities in the appearance of cancer cells between primary tumors and their corresponding metastases. Lymph nodes from patients with other breast cancer subtypes also revealed senescent tumor cells within metastatic lesions. These results suggest a potential role for senescent breast tumor cells during metastatic progression, raising the question as to whether targeting senescent tumor cells might represent a novel avenue for improved treatment of breast and other cancers.

**Abstract:**

Oncogene-induced senescence is thought to constitute a barrier to carcinogenesis by arresting cells at risk of malignant transformation. However, numerous findings suggest that senescent cells may conversely promote tumor growth and metastatic progression, for example, through the senescence-associated secretory phenotype (SASP) they produce. Here, we investigated the degree to which senescent tumor cells exist within untreated human primary breast carcinomas and whether the presence of senescent cancer cells in primary tumors is recapitulated in their matched lymph node metastases. For the detection of senescence, we used SA-β-galactosidase (SA-β-gal) staining and other senescence markers such as Ki67, p21, p53, and p16. In patients with invasive luminal A and B breast carcinomas, we found broad similarities in the appearance of cancer cells between primary tumors and their corresponding metastases. Analysis of lymph nodes from patients with other breast cancer subtypes also revealed senescent tumor cells within metastatic lesions. Collectively, our findings show that senescent tumor cells exist within primary breast carcinomas and metastatic lesions. These results suggest a potential role for senescent breast tumor cells during metastatic progression and raise the question as to whether the targeting of senescent tumor cells with anti-senescent drugs might represent a novel avenue for improved treatment of breast and other cancers.

## 1. Introduction

Hallmark characteristics of cellular senescence include withdrawal from the cell cycle, macromolecular damage, deregulated metabolism, and the production of a senescence-associated secretory phenotype (SASP), in which a variety of factors are secreted that can influence the behavior of neighboring and immune cells [1,2,3,4,5,6]. The induction of senescence as a consequence of oncogene activation is considered to represent a barrier to tumorigenesis [1,7]. Consistently, senescent cells have been observed within premalignant tumors [8,9,10,11,12,13]. For cancer development, it has therefore been suggested that the precursors of tumor cells need to circumvent or escape from oncogene-induced senescence and gain the ability to proliferate while expressing activated oncogenes [14].

The tumor suppressor signaling pathways Arf-p53 and pRB-16INK4a facilitate the induction of senescence in response to oncogenic stimuli [1,15]. Evasion of senescence and escape from growth arrest is fostered by cell-autonomous mechanisms that involve genetic or epigenetic changes. As an example, functional defects in Arf-p53 and pRB-p16INK4a signaling can prevent the induction of senescence and promote malignant progression [12,16,17,18]. Furthermore, tumor-infiltrating immune cells can counter senescence through non-cell autonomous mechanisms that protect proliferating tumor cells from senescence or enable already arrested cells to escape from oncogene-induced senescence, thus sustaining tumor growth [19].

Senescence can be induced in tumor cells [7], suggesting that senescence is more than just a hurdle to tumorigenesis during the development of cancer. For example, conventional cancer therapies can induce senescence in tumor cells [20]. Mechanistically this can be due to p53 reactivation [21,22] or loss of Skp2 [23] in tumor cells or can be caused by T-helper cells that invade the tumor and secrete cytokines such as IFN-γ and tumor necrosis factor (TNF) that stimulate senescence [24]. The induction of senescence can lead to regression of the tumor and may be coupled with an inflammatory response that induces immune cells to destroy and clear the senescent tumor cells [2,22,25].

Paradoxically, senescent cells within tumors can promote the growth and progression of non-senescent cancer cells. Many SASP components are pro-tumorigenic growth factors, matrix metalloproteinases (MMPs), and cytokines that are known to stimulate the aggressive behavior of cancer cells in vitro [26,27,28,29,30]. Through their SASP, senescent cells can promote the progression of both precancerous cells and established cancer cells in mouse xenograft models [26,31]. For example, when breast cancer cells that express constitutively active HER2 enter senescence, the SASP they produce stimulates the metastatic progression of non-senescent tumor cells and inhibits the immune clearance of senescent cells [32]. In B-Raf mutated papillary thyroid carcinomas, senescent cancer cells promote collective invasion of senescent and non-senescent cancer cells and foster metastatic progression through their SASP [30]. In colorectal cancers, senescent tumor cells protect non-senescent cancer cells from immune cells by building a cytokine shield out of SASP components [6]. In the context of prostate cancer, loss of tissue inhibitor of metalloproteinases-1 (TIMP1) causes activation of MMPs and thereby reprogramming of the SASP into a SASP that fosters metastasis [33]. MMPs in the SASP can also cleave NKG2D ligands, which suppresses NK cell-dependent immune surveillance and enables senescent cells to evade immune clearance [34]. The latter examples illustrate the particular importance of MMPs, which are major components of SASPs, in promoting the progression of malignancies by either facilitating migration and invasion or enabling cancer cells to evade immune clearance.

Although SASP components may promote malignant progression through paracrine signaling, the composition of SASPs is complex, and not all SASP factors promote cancerogenesis [1], and some SASP components stimulate the elimination of senescent cells by the immune system [2,5,22]. Thus, the recruitment of immune cells and the consequences of the immune response to senescent cells is determined by the exact composition of the SASP. Furthermore, the effect of individual SASP components depends on the cellular microenvironment and tissue-specific context [5,32]. Moreover, senescence is not always irreversible. Molecular mechanisms exist that enable tumor cells to escape from senescence and cancer cells that exit senescence acquire a more aggressive phenotype [35].

The ability of senescence to act as a barrier to tumor progression on the one hand but also to promote tumor initiation and metastasis on the other has been demonstrated in murine xenograft and transgenic models [2,22,27,30,31,32,33,34,35,36,37]. Senescent tumor cells have been observed within several therapy-naïve human carcinomas [30,38]. In human therapy-naïve breast carcinomas, senescent tumor cells exist, and their presence is dependent on the molecular subtype [38]. However, the possible existence of senescent tumor cells within breast cancer metastases has not been explored to date. In the present study, we investigated senescence-associated β-galactosidase (SA-β-gal) activity within primary luminal breast cancer samples and their matched sentinel lymph node metastases from patients who had not been treated with neoadjuvant therapy or radiotherapy. We also investigated whether senescent tumor cells exist within metastatic lesions from patients with other breast cancer subtypes.

We found similarities in the appearance of senescent cancer cells between primary tumors and their corresponding metastases within luminal A and B breast carcinomas. Analysis of lymph nodes from patients with other breast cancer subtypes also revealed senescent tumor cells within metastatic lesions. As senescent cancer cells avoid recognition by the immune system through MMPs in their SASP [34], these observations might reflect the ineffective elimination of senescent breast cancer cells by the immune system due to SASP-mediated suppression of immune surveillance and might also explain why breast carcinomas show an inefficient response to cancer immunotherapies. Taken together, our observations raise the question as to whether the targeting of senescent tumor cells with senolytic or senomorphic drugs might be beneficial for improving the treatment of breast and other cancers.

## 2. Materials and Methods

### 2.1. Patient Samples

Tissue samples from 67 lymph node metastases and tissue of the breast carcinomas from 11 patients with primary early breast cancer were collected in the Institute of Pathology at the University Medical Center of the Johannes Gutenberg University in Mainz between January 2015 and 2018. All patients were female and received a modified radical mastectomy or breast-conserving surgical therapy with sentinel lymph node resection. Patients treated with neoadjuvant therapy prior to surgery were excluded from this study. All included lymph node metastases and primary breast carcinomas were pathologically evaluated at the Institute of Pathology at the University Medical Center in Mainz. The pathological characteristics of the samples were obtained from the original pathology reports and included the histological tumor type, histological grade, estrogen receptor status (ER), progesterone receptor status (PR), HER2-2-neu status (HER2), and the proliferation index (Ki67). Staging employed the TNM classification of malignant tumors (7th Edition 2009) and the WHO classification of breast cancer [39]. The Nottingham histologic score system (the Elston-Ellis modification of the Scarff-Bloom-Richardson grading system) [40] was used for histological evaluation.

In this study, we used 11 frozen primary tumor samples and 67 frozen metastatic tissues donated by patients who had previously been diagnosed with primary breast cancer following histological evaluation of preoperative needle core biopsies. For the primary tumor samples, the margins of the surgical specimens were delimited with ink during the intraoperative assessment, and the part of the tumor with the nearest surgical margin was frozen and collected immediately after intraoperative diagnostic evaluation. The intraoperative assessment of the sentinel lymph node (SLN) served as an intraoperative diagnostic evaluation of metastases. Lymph nodes suspected to contain tumor tissue were frozen. The intraoperative histological analysis involved taking two consecutive frozen sections from the primary tumor and lymph node samples. One section was used for intraoperative pathological assessment following staining with hematoxylin and eosin (H&E). The second cryosection was stored for subsequent analysis.

### 2.2. Immunohistochemical Detection and Scoring Method

Formalin-fixed and paraffin-embedded lymph node metastases and primary tumor tissues were used for immunohistochemical staining. Standard methods were used for immunohistochemistry and employed an immunostainer (Techmate 500; Dako, Glostrup, Denmark) according to the manufacturer’s instructions. Specific antigens were detected using the monoclonal antibodies indicated in brackets: ER (1D5), PR (1A6), Ki67 antigen (MIB-5), p21^Cip1/Waf1^ (Sx1118), and p53 (DO-7). These antibodies were purchased from Dako (Glostrup, Denmark). Staining was visualized using the avidin-biotin complex (ABC) method, with AEC (3-amino-9-ethylcarbazol) as the chromogen. Expression of p16^INK4A^ was analyzed by using CINtec^TM^ p16 (E6H4) (Roche Diagnostics, Basel, Switzerland). The Hercept-test and HER2 FISH pharmDx^TM^ Assay Kit (Dako, Glostrup, Denmark) were used to determine HER2 status.

The Ki67 proliferation index and protein expression of p16^INK4A^, p21^Cip1/Waf1^, and p53 were quantified on the basis of percentage positivity in at least 500 neoplastic cells counted in the tumor area. Negative, low, and high protein expression levels of p16^INK4A^, p21^Cip1/Waf1^, and p53 were defined as follows: p16 and p53 negative < 1% positive cells, p16 and p53 low (+): score 1+ or 2+ ≤ 50% pos. cells or score 3+ or 4+ ≤ 10% pos. cells. p16 and p53 high (++): score 1+ or 2+ > 50% pos. cells or score 3+ or 4+ > 10% pos. cells. p21 negative < 1% positive cells, p21 low (+) < 5% pos. cells, p21 high (++) ≥ 5% pos. cells. Ki67 negative < 1% positive cells, Ki67 low < 40% pos. cells, Ki67 high ≥ 40% pos. cells. For the Ki67 proliferative index and for p16^INK4A^, p21^Cip1/Waf1^, and p53 expression, only nuclear reactivity was taken into account. For the assessment of ER and PR staining, strong nuclear staining in one or more tumor cells was considered positive. The immunohistochemical intensity and the percentage of positive cells were used to determine the ER/PR status. HER2 overexpression (3+) was defined as complete and strong membrane staining in more than 10% of the tumor cells. Amplification of HER2 is defined by a HER2:Cep17 ratio of more than 2.0 [41].

### 2.3. St. Gallen Risks Groups

The St. Gallen international breast cancer conference guidelines (2013) were used to classify the subtypes of breast cancer used in this study [42]. Luminal-A breast cancer was defined as ER+/PR+, Ki67 low (less than or equal to 20% positively stained tumor cells), and HER2 negative. Luminal-B was defined as ER+, PR−/low, or Ki67 high (more than 20% tumor cell positivity), and HER2+/−. HER-2-positive tumors were defined as ER−, PR−, and HER2+. Tumors that were negative for ER, PR, and HER2 were classified as triple-negative breast cancer.

### 2.4. Senescence-Associated β-Galactosidase Activity and Scoring Method

Senescence-associated β-galactosidase (SA-β-gal) staining was carried out as described previously [43]. After airdrying overnight, frozen sections were fixed with 2% formaldehyde and 0.2% glutaraldehyde for 1 h, with all procedures being carried out at room temperature. Sections were then incubated in β-galactosidase staining solution for 24 h at 37 °C. 5-bromo-4-chloro-3-indolylP3-D-galactoside (X-Gal) was purchased from Carl Roth GmbH, Karlsruhe, Germany. Staining with 4′,6-Diamidino-2-phenylindole (DAPI, Sigma Aldrich, Taufkirchen Germany) was used to visualize nuclei. Negativity for SA-β-gal in samples was defined as either no SA-β-gal staining being present or only non-tumor cells being SA-β-gal positive. Intermediate positivity for SA-β-gal (+) was defined by strong cytoplasmatic staining in more than 1% and less than 50% of the tumor cells in the sample. The SA-β-gal high positive (++) samples exhibited strong cytoplasmatic staining in more than 50% of the tumor cells.

### 2.5. Statistical Analysis

Fisher’s exact test was performed using the Microsoft Excel software package. Statistical significance was defined as a *p*-value of <0.001.

## 3. Results

### 3.1. Constitution of the Patient Sample Collection

To investigate the existence of senescent tumor cells within breast cancer metastases, tissue sections of the SLN from 67 patients with early breast cancer were collected. To ensure that senescence within tumor cells was not induced by previous treatments such as chemo- or radiotherapy, only lymph nodes taken from patients prior to systemic therapy were included in this study. The histological subtypes, grading, and molecular subtypes of the sample collective used in this study are summarized in Table 1 and Table 2, with subtype classification following the St. Gallen molecular subtype definitions [42]. Of the 67 patients included in this study, SLN samples from only 63 patients contained sufficient tumor material to be included in the analyses.

### 3.2. SA-β-gal-Positive Tumor Cells Exist within Primary Luminal Breast Carcinomas and in Their Matched Sentinel Lymph Node (SLN) Metastases

**Figure 1:**
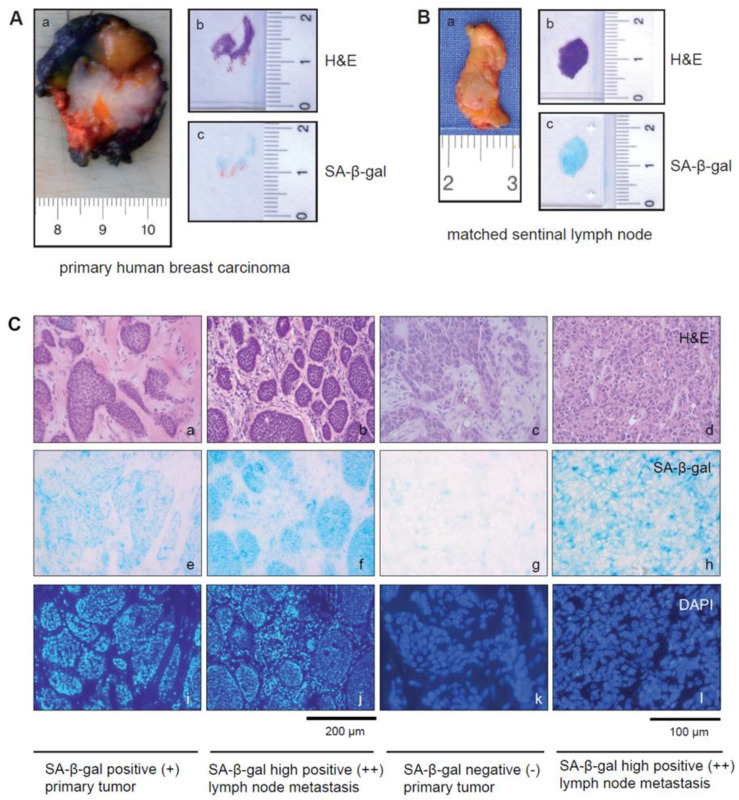
SA-β-gal staining for detection of senescent cells within primary breast cancer and/or lymph node metastases. (**A**(**a**)) Representative macrophotos of a primary human breast carcinoma with ink on margins and (**B**(**a**)) the matched sentinel lymph node. (**A**(**a**–**c**)) Margins of the surgical specimen were marked with ink, and the part of the tumor with the next surgical margin was frozen. Two successive frozen sections were generated from (**A**(**b**,**c**)) tumor tissue or (**B**(**b**,**c**)) from lymph node metastases immediately after surgery. (**A**(**b**),**B**(**b**)) One frozen section was stained with hematoxylin and eosin (H&E), (**A**(**c**),**B**(**c**)) A second proximate section of the frozen breast tissue and lymph node metastases from each case was obtained and collected for the detection of SA-β-gal activity. (**C**(**a**–**d**)) Consecutive H&E-stained frozen sections corresponding to sections shown in (**e**–**h**). (**e**–**h**) SA-β-gal staining of the frozen tissue sections of primary breast carcinomas (**a**,**c**,**e**,**g**,**i**,**k**) and the matched SLN metastases (**b**,**d**,**f**,**h**,**j**,**l**) from two patients. Note the differences in the number of SA-β-gal positive tumor cells present in the primary tumors and the matched SLN metastases. (**a**,**b**) patient 3 and (**c**,**d**) patient 8 (Table 3). (**a**) SA-β-gal positive (+); (**b**,**d**) SA-β-gal high positive (+ +); (**c**) SA-β-gal negative (−). (**i**–**l**) DAPI-staining of the sections shown in (**e**,**f**,**g**,**h**). Bars: 200 µm or 100 µm as indicated.

Previously we reported that senescent cells exist in primary invasive breast carcinomas [38]. To address whether senescent tumor cells also exist in metastatic breast cancer lesions, tissue sections from primary tumors and their lymph node metastases were collected. For both primary tumor and SLN samples, two consecutive frozen sections were taken immediately after surgery (Figure 1A,B). One was stained intraoperatively with H&E, and the other was used subsequently for SA-β-gal staining (Figure 1A(b,c),B(b,c)). SA-β-gal staining was observed in the primary tumor samples and in the majority of the SLN metastatic lesions. Cytoplasmic staining was generally homogeneous but differed in amount and intensity from sample to sample (Figure 1C(e–h)), with the vast majority of SA-β-gal positive cells being clearly identifiable as tumor cells. However, some patient samples contained few, if any, SA-β-gal positive tumor cells (Figure 1C(g)).

**Figure 2:**
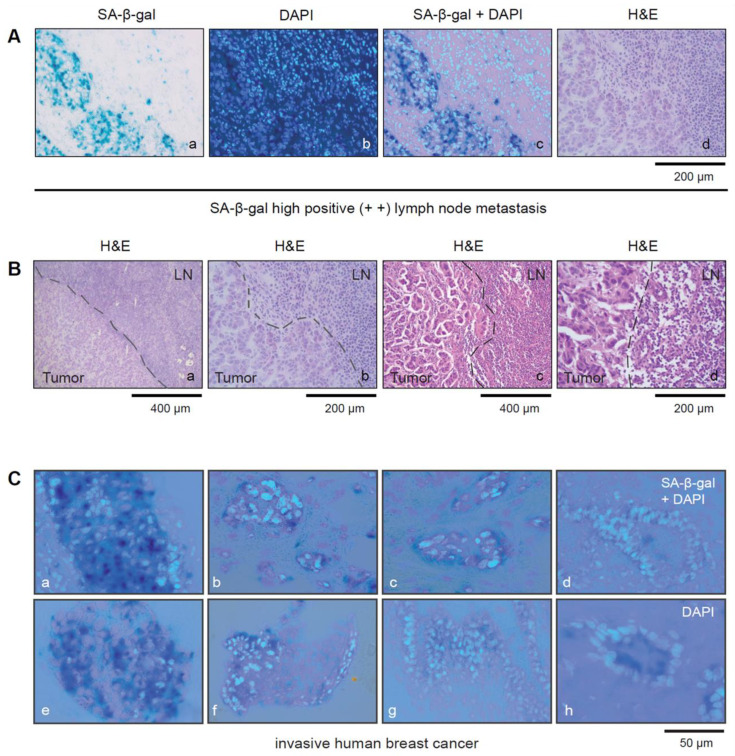
(**A**) Tumor cells within SLNs can be distinguished from immune cells by SA-β-gal staining (**a**–**d**). (**a**) SA-β-gal staining; (**b**) DAPI staining; (**c**) SA-β-gal/DAPI staining overlay (**d**) H&E staining. Bar: 200 µm (**B**(**a**,**d**)) Pictures showing lymph node metastasis from two different patients with SA-β-gal high positive SLN metastases (**a**,**b**) patient 3 and (**c**,**d**) patient 8 (see Table 3). (**B**(**a**,**b**)) shows corresponding H&E stained sections of patients 3 shown in (**A**(**a**–**d**)) and (**c**,**d**) shows patient 8 with an SA-β-gal positive SLN metastasis (see Figure 1C(c,d)). Bars: 200 µm or 400 µm as indicated (**C**(**a**–**h**)) Magnified pictures showing representative image sections from some of the SA-β-gal-stained breast carcinomas. SA-β-gal/DAPI overlay Bar: 50 µm.

Notably, the immune cells within the SLNs showed no or only very weak SA-β-gal staining and were defined as SA-β-gal negative, whereas tumor cells could be clearly identified by strong cytoplasmic SA-β-gal positivity in most of the samples (Figure 2A–C).

### 3.3. SA-β-gal-Positive Tumor Cells from Luminal Breast Carcinomas Exist in Tumor Cell Clusters within Lymph Vessels in the Perinodal Tissue

**Figure 3:**
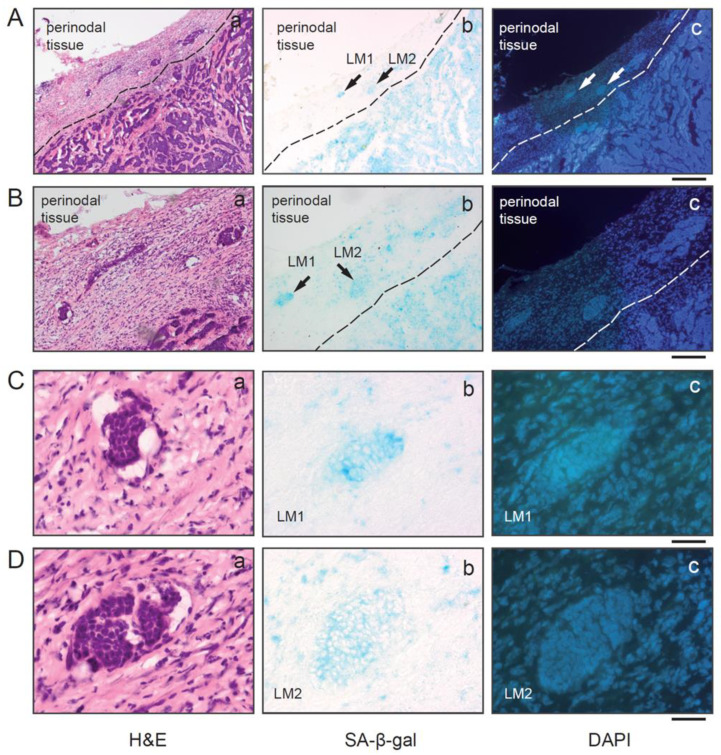
SA-β-gal positive tumor cells are present within luminal breast cancer cell clusters in lymph vessels. (**A**) Serial sections of a SLN with metastatic lesions and tumor cell clusters (LM) within two lymphatic vessels within the perinodal tissue (LM1+LM2) Bar: 400 µm. (**B**) Enlarged view of the tumor cell clusters within the lymphatic vessels. Bar: 200 µm (**C**) LM1 and (**D**) LM2. Bars: 50 µm (**a**) Hematoxylin and eosin (H&E) staining of the frozen section; (**b**) SA-β-gal staining; (**c**) DAPI staining.

Closer inspection of the tissue sections from the SLN revealed the existence of multiple tumor cells within lymph vessels in the perinodal tissue that were SA-β-gal positive (Figure 3A–D). This observation suggests that either senescent luminal breast cancer cells can disseminate and exist within the circulation and reaches distant organs or that disseminated tumor cells become senescent after dissemination.

### 3.4. Correlation between SA-β-gal Staining and Other Senescence Markers within Primary Luminal Breast Carcinomas and Their Matched SLN Metastases

We obtained primary breast cancer samples from 11 treatment-naive patients together with matched SLNs. However, only 10 of these 11 patient samples contained tumor tissue in the frozen tissue section and were therefore eligible for inclusion in this study. All of these 10 samples were from patients diagnosed with luminal molecular breast cancer subtypes. Eight of the patients had luminal A, and two patients had luminal B breast carcinomas (Table 3). No tissue sections of primary breast carcinomas from patients with HER2+ or triple-negative breast cancer (TNBC) could be included in the study since these patients had already received neoadjuvant treatment prior to the collection of the primary tumor samples.

We observed SA-β-gal positive tumor cells in the primary tumors and in their matched SLN metastases in eight patients, while no SA-β-gal positive tumor cells could be detected in the samples from one patient (Table 3). Interestingly, for two patients (Table 3, patients 3 and 8), we observed differences in the amount of SA-β-gal positive tumor cells between primary tumors and the matched SLN metastases (Table 3, Figure 1C), with a higher incidence of SA-β-gal positive tumor cells in the metastases compared to the primary tumor.

Senescent cells are typified by cell cycle arrest, and therefore SA-β-gal-positive cells would be expected not to proliferate. Nevertheless, several studies report data showing that cancer cells can escape from senescence and re-enter the cell cycle [16,17,35]. To investigate whether SA-β-gal-positive tumor cells in our samples were growth arrested, we analyzed the expression of the proliferation marker Ki67 as well as additional proteins that are inhibitors of cell cycle progression such as p53, p16^INK4A^, and p21^Cip1/Waf1^ within SA-β-gal positive tumor cells as described previously [38]. To assess the proliferation status of SA-β-gal positive tumor cells, we first quantified the percentages of Ki67 positive tumor cells within all primary breast cancers and SLN metastases samples. For this purpose, we used paraffin sections from a piece of the tumor and the lymph node that corresponded exactly with the position in the tumor and the lymph node from which the frozen sections for SA-β-gal staining had been taken. However, metastatic lesions in the SLN from one patient were not large enough for tumor cells to be found on subsequent sections of the LNs (Table 3, patient 2).

The percentages of Ki67 positive tumor cells within all primary luminal A breast tumors and their matched SLN metastases were in a range of approximately <5 and 30% and 25–30% for the luminal B cases (Table 3). Seven out of ten primary tumors and eight out of ten SLN metastases were either SA-β-gal positive or SA-β-gal high positive. Stratification of tumors and SLN metastases on the basis of Ki67 low (Ki67 < 40%) or Ki67 high (Ki67 > 40%) staining revealed an inverse correlation between SA-β-gal positivity and Ki67 positivity. This observation is in accordance with our previous findings, which showed that the vast majority of luminal breast carcinomas (93.7%) contained 5–35% Ki67 positive tumor cells and that SA-β-gal positivity is inversely correlated with Ki67 positivity, supporting the notion that senescent SA-β-gal positive tumor cells are cell cycle arrested [38].

**Figure 4:**
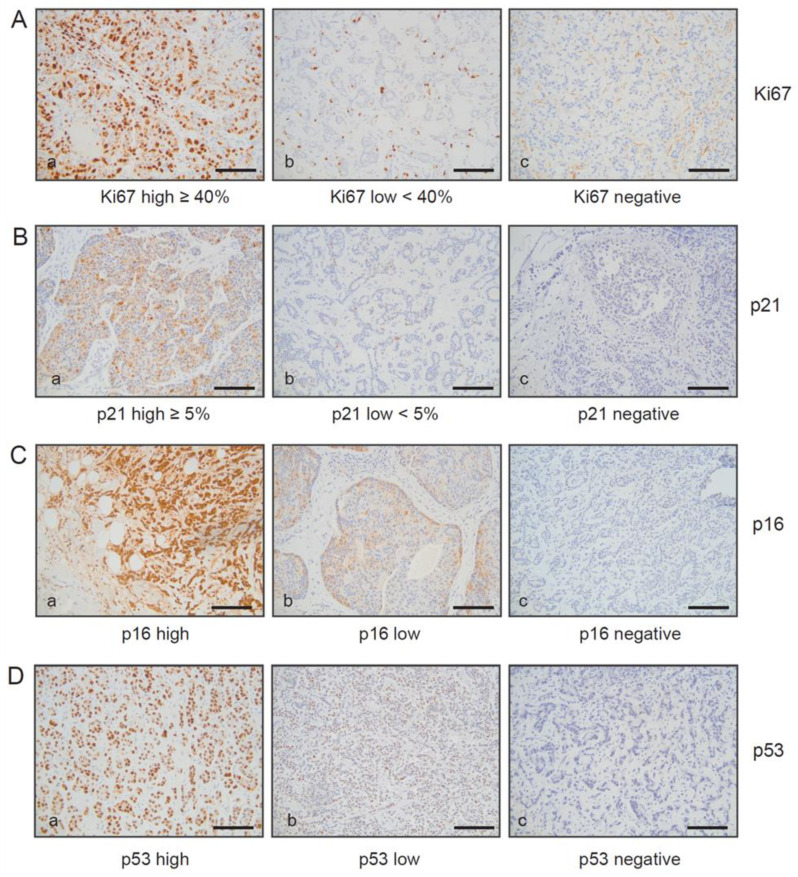
Immunohistochemical analysis of Ki67, p16^INK4A^, p21^Cip1/Waf1^, and p53 expression within primary breast carcinomas and in their matched lymph node metastases. Serial sections of primary breast carcinomas and their matched metastasis were stained (**A**) anti-Ki67 antibodies, (**B**) anti-p21 ^Cip1/Waf1^ antibodies, (**C**) anti-p16^INK4A^, and (**D**) p53 antibodies. Represent examples are shown: (**A**–**D**) (**a**): Ki67, p21 ^Cip1/Waf1^, p16 ^INK4A^, and p53 high; (**A**–**D**) (**b**): Ki67,p21 ^Cip1/Waf1^, p16 ^INK4A^, and p53 low; (**A**–**D**) (**c**): Ki67, p53, p16 ^INK4A^, and p21 ^Cip1/Waf1^ negative. Protein expression of Ki67 and p21^Cip1/Waf1^ were quantified on the basis of percentage positivity in at least 500 neoplastic cells counted in the tumor area. Protein expression of p16^INK4A^, p21 ^Cip1/Waf1^, and p53 negative, low, and high expression were defined as follows: p16 and p53 negative < 1% positive cells, p16 and p53 low (+): score 1+ or 2+ ≤ 50% pos. cells or score 3+ or 4+ ≤ 10% pos. cells. p16 and p53 high (++): score 1+ or 2+ > 50% pos. cells or score 3+ or 4+ > 10% pos. cells. p21 negative < 1% positive cells, p21 low (+) < 5% pos. cells, p21 high (++) ≥ 5% pos. cells. Ki67 negative < 1% positive cells, Ki67 low < 40% pos. cells, Ki67 high ≥ 40% pos. cells. For Ki67, p21 ^Cip1/Waf1^, p16 ^INK4A^, and p53, only nuclear reactivity was taken into account. Bar: 200 µm.

Given the dependence of the senescence program on the tumor suppressor pathways Arf-p53 and pRB-p16^INK4A^ [1,15], we investigated the degree to which SA-β-gal-positive tumor cells within the primary breast cancer and their related metastases samples also express p53 and p16^INK4A^. To this end, the collective of SA-β-gal positive primary luminal breast cancers and their matched metastases were immunohistochemically stained for p53 and p16^INK4A^ expression (Table 3). We observed moderate p53 expression in seven of the eight primary luminal A tumors and their matched metastases, with the exception of one metastasis that displayed high p53 expression and one primary tumor that was p53 negative. The two luminal B breast carcinomas and their matched SLN metastases also displayed high p53 expression (Table 3). Consistent with these results, we previously reported low p53 expression in most luminal A and B breast carcinomas, indicating that the Arf-p53 pathway is likely to still be functional in the majority of these breast tumors [38]. Of note, the only patient with SA-β-gal negative primary tumor and negative SLN metastasis had few (<1%), if any, p53-positive and p21 negative tumor cells (Table 3), indicating loss of Arf-p53 function. Representative images for Ki67, p21 ^Cip1/Waf1^, p16 ^INK4A^, and p53 are shown in Figure 4A–D.

### 3.5. SA-β-gal-Positive Tumor Cells Are Present in Primary Breast Carcinomas, but No SA-β-gal-Positive Epithelial Cells Were Found in the Normal Breast Tissue Surrounding the Tumor

Under normal physiological conditions, senescent cells are subject to immune surveillance by various components of the immune system. Senescent cells attract and activate immune cells and serve as immunogenic targets for elimination by the immune system. Normally, the deletion of senescent cells allows the regeneration of the tissue in which they reside. However, in advanced age or under pathophysiological conditions that either lead to impaired immune function or when senescent cells develop strategies to evade immune surveillance, senescent cells may accumulate in normal tissues. Under such conditions, the positive cell-autonomous role of senescence could be abrogated by the negative effects of senescent cells with their SASP on surrounding cells. A decrease in immune function in advanced aging or under pathophysiological conditions leading to decreased immune system function has been associated with the development of cancer [44].

In the context of aging, the extent to which the immune system is involved in regulating the number of senescent cells and whether age-related impairment of immune function contributes to the accumulation of senescent cells in the elderly is unknown [25]. Theoretically, the accumulation of senescent cells in breast tissue due to either aging or impaired immune surveillance could promote the development of breast cancer and later its progression. To investigate whether senescent cells are present in normal human breast tissue and whether their occurrence correlates with age, we examined the normal tissue surrounding the tumor for the presence of senescent non-tumor cells.

**Figure 5:**
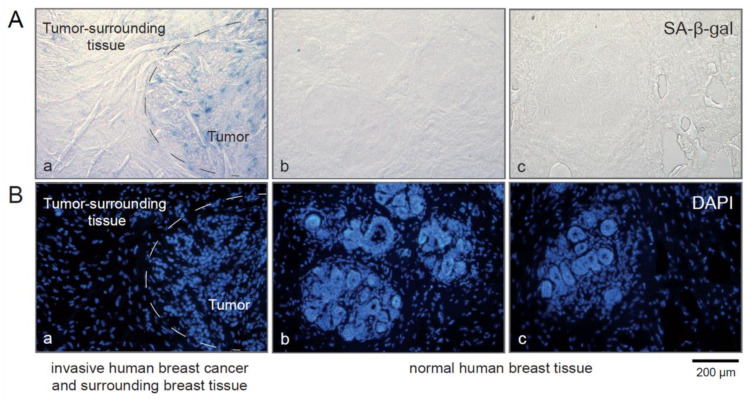
SA-β-gal positive cancer cells are present within primary breast carcinomas, but the normal breast tissue surrounding the tumor does not contain SA-β-gal-positive cells. (**A**,**B**) (**a**): human breast carcinoma + surrounding normal tissue (**A**,**B**) (**b**,**c**) normal human breast tissue. (**A**) (**a**–**c**): SA-β-gal staining, (**B**) (**a**–**c**): DAPI. Bar: 200 µm.

The age of the patients from whom we collected primary tumors and matched SLN metastases ranged from 43 to 83 years (see Table 4). We did not observe SA-β-gal positive cells within the normal breast tissue surrounding the tumor (Figure 5A,B). From this observation, we conclude that senescent cells do not accumulate in breast tissue even at older ages.

### 3.6. Detection of SA-β-gal Positive Breast Cancer Cells within Lymph Node Metastases Derived from Different Molecular Breast Cancer Subtypes

In our previous study, we reported that the existence of senescent breast cancer cells distinguishes molecular breast cancer subtypes [38]. To investigate whether the existence of senescent breast cancer within SLN metastases can also be correlated with the molecular breast cancer subtypes, we performed SA-β-gal stainings on the remaining SLNs that we collected (Table 2). Since triple-negative and HER2+ breast cancer patients received neoadjuvant treatment, no tissue sections from primary TNBC or HER2+ breast carcinomas could be collected for this study. However, the patients with TNBC or HER2+ breast carcinomas underwent a SLN resection prior to neoadjuvant therapy. Thus, only these treatment-naive SLNs were used for the detection of senescent breast cancer cells. For SLNs taken from patients with HER2+ breast carcinomas, we observed SA-β-gal positive tumor cells (SA-β-gal high (+ +) and low (+)) in 100% of the SLN metastases, and high SA-β-gal positivity (+ +) in 87.5% of the samples (Table 5). In patients with luminal breast carcinomas, we observed SA-β-gal positive tumor cells (SA-β-gal high (+ +) and low (+)) in their SLN metastases in 88.3% of cases (Table 5). The TNBC SLN metastases also contained high numbers of SA-β-gal positive tumor cells, which is notable because in a previous study few, if any SA-β-gal positive tumor cells were observed in primary tumor samples from TNBC patients [38].

### 3.7. Ductal and Lobular Carcinomas Differ in the Incidence of SA-β-gal Positive Tumor Cells within SLN Metastases

Of the 52 luminal breast carcinoma SLNs analyzed in this study, 46 cases were invasive ductal carcinomas (IDC), and 6 were invasive lobular carcinomas (ILC). Six SLN metastases did not include SA-β-gal positive cancer cells (Table 6). Interestingly, a significant difference in the distribution of SA-β-gal positive samples was observed between IDC and ILC. Specifically, 2/46 SLN metastases from IDCs were negative for SA-β-gal staining, whereas 4/6 ILC samples were negative for SA-β-gal staining (Table 6). Although the number of ILC samples in this analysis is low, these data suggest that ILC are likely to contain substantially fewer SA-β-gal positive tumor cells compared to IDC.

## 4. Discussion

Cellular senescence is considered to be a barrier to oncogene-induced carcinogenesis [1,20]. A few early studies reported senescent cells in premalignant human naevi and colon adenomas, but in comparison, no or significantly reduced numbers of senescent cells were found in malignant melanomas and adenocarcinomas. These studies concluded that cellular senescence might not be relevant in the context of advanced cancers and their metastases [8,9,10,11,12,13]. However, later studies clearly showed that in the context of untreated invasive human carcinomas from colon and BRAF-mutated thyroid cancer patients, significant numbers of senescent cells could be present in the primary tumor [6,30]. We have previously reported that senescent tumor cells also exist within human breast tumors [38]. To follow up on this finding, in the present study, we evaluated the presence of senescent cancer cells within primary invasive breast carcinomas and their matched SLN metastases. Our results show that the majority of the primary luminal tumors and their matched metastases contain large numbers of senescent cells, which was also true for SLNs taken from pre-therapy patients with HER2+ and TNBC breast cancers. These observations are consistent with our previous work in which we observed high numbers of SA-β-gal positive tumor cells within HER2+ primary tumors [38]. Taken together, our findings suggest that cellular senescence is a feature of metastases from all breast cancer molecular subtypes.

Senescent tumor cells can contribute to metastatic dissemination in a number of ways, including the fostering of tumor cell invasion [29] and metastatic progression [30] and protecting against anti-tumor immune responses [6]. Mechanistically, the SASP produced by senescent cells in cancer tissue plays a major role in increasing the malignant potency of cancer cells [31]. For example, cancer therapies can induce senescence in both the tumor microenvironment and in cancer cells, and the SASPs that are produced as a consequence can negatively impact treatment efficacy in a number of ways and cause cancer progression [45]. Consistently, the targeting of therapy-induced senescent cells by senolytics or by suppressing the secretion of SASPs has been shown to improve cancer treatment outcomes [46]. For example, in ovarian cancer, platinum-containing chemotherapy induces cellular senescence and an associated SASP that promotes cancer stem cell formation and promotes tumor recurrence. This can be suppressed by treatment with the NAMPT inhibitor FK866 [47]. In prostate cancer, radiation treatment leads to the induction of senescence in prostate cancer cells and fibroblasts and the release of an NFkB-driven pro-inflammatory SASP. The mTOR-inhibitor rapamycin prevents the expression of the SASP induced by radiation treatment and thereby inhibits tumor progression [48,49]. These examples illustrate that targeting therapy-induced senescent cells can improve cancer treatment.

In BRAF-mutated papillary thyroid carcinomas, senescent tumor cells promote the collective invasion of senescent and non-senescent tumor cells via their SASP. Furthermore, the senescent tumor cells induce anoikis resistance in non-senescent tumor cells during the passage through the blood and lymph flow, helping non-senescent tumor cells to survive within the circulatory system [30]. Collective invasion is a feature of the majority of human breast carcinomas [50]. Thus, it is conceivable that senescent cells also support the collective invasion and the metastatic process in breast carcinomas through their SASP in a manner similar to that observed in papillary thyroid carcinomas, in particular in breast cancer subtypes with a high incidence of senescent tumor cells such as luminal A and HER2+ [38]. Furthermore, cell cycle-arrested senescent cells are likely to be less susceptible to anti-cancer drugs and are not recognized by the immune system. They might therefore be able to remain dormant for prolonged periods but eventually regain proliferative capacity and form metastases [51].

Interestingly, the SASP of senescent cells transformed by constitutive HER2 signaling inhibits the clearance of senescent cells and exerts pro-metastatic effects leading to breast cancer progression in mice xenografts [32]. Taken together, these observations suggest that further investigations should focus on defining the constitution of the SASP produced by senescent cells in human luminal A and HER2+ breast carcinomas and determining how different SASP components foster cell survival, anoikis resistance, dormancy, drug resistance, and protection from the anti-tumor immune response.

Luminal A breast carcinomas are well differentiated, have a low Ki67 index, and are associated with a good prognosis. However, these tumors are characterized by late recurrence and the formation of metastases many years after apparently successful treatment. Thus, these tumors seem to disseminate early, and tumor cells stay dormant over a long period of time. The dormant properties of these cells may be linked to senescence. Cancer cells can exit senescence through cell and non-cell autonomous mechanisms such as alterations within the tumor suppressor pathways p53-p21^Cip1/Waf1^ and pRB-p16^INK4A^ or through the infiltration of immune cells, such as myeloid cells, that enable already arrested cells to escape from oncogene-induced senescence, and thus sustain tumor growth [19]. Following escape from therapy-induced senescence, tumor cells acquire stem-like properties and a more aggressive phenotype, which is paralleled in breast cancer patients with recurrent tumors [35].

Recurrence within luminal breast cancer patients is strongly associated with resistance to endocrine treatment [52]. SASP components produced by senescent luminal breast cancer cells could conceivably lead to the activation of signaling pathways such as Wnt and STAT3 that are associated with resistance to endocrine treatment [53,54], or promote the survival of disseminated tumor cells in the presence of endocrine treatment over long periods of time. Further studies are required to understand the role of senescent luminal A tumor cells and their SASPs in resistance to endocrine therapies and whether targeting senescent luminal breast carcinoma cells might be useful to overcome endocrine resistance and avoid late recurrence.

Previously we have reported that few, if any, SA-β-gal positive tumor cells exist within primary human TNBCs [38]. Surprisingly, we observed SA-β-gal positive tumor cells within all of the TNBC metastases analyzed. A possible explanation for the high numbers of SA-β-gal positive tumor cells within SLN metastases from TNBC patients might be that these tumor cells have a higher mutational burden and might therefore be better detected by the immune system and thus more accessible to immune surveillance. Tumor-infiltrating T-helper 1 cells can induce senescence in tumor cells through secretion of the cytokines IFN-γ and tumor necrosis factor (TNF) [24]. Thus, it is conceivable that senescence is induced in TNBC cells when they encounter CD4+ T-cells in the SLNs. It can be speculated that these tumor cells must activate escape mechanisms to leave the LNs and to form metastases at distant organ sides. Escape from therapy-induced senescence is associated with a stem-like phenotype and more aggressive behavior [35]. Maybe this mechanism also represents a selection process within the LNs for immune evasion and escape from immune cell-induced senescence ending up in aggressive, stem-like TNBC cells. Interestingly, certain tumor-infiltrating immune cells can oppose senescence in murine models [19]. It was shown that CD11b(+)Gr-1(+) myeloid cells can protect proliferating tumor cells from senescence or enable already arrested cells to escape from oncogene-induced senescence by antagonizing senescence in a paracrine manner through interfering with the SASP of the tumor and secretion of interleukin-1 receptor antagonist (IL-1RA) [19]. Possibly, myeloid-derived suppressor cells might be involved in the escape from immune cell-induced senescence and, as a consequence, in metastatic progression.

A limitation of this study is the relatively low number of SLN metastases analyzed, as well as the fact that we could only make a direct comparison between primary tumors and SLN metastases in the context of luminal A and B breast cancers. The widespread neoadjuvant treatment of breast cancer patients strongly restricts the possibility of collecting untreated primary tumors and their matched metastases. Nevertheless, it is notable that significantly fewer ILC SLN samples contained senescent cells compared to SLN from IDC, despite the relatively low number of ILC samples in this analysis (Table 6). Although IDCs and ILCs are histologically and clinically different, ILC is nevertheless treated similarly to IDC [55]. However, ILC is less responsive to systemic chemotherapy compared to IDC [55]. Differences in the number of SA-β-gal positive tumor cells between ILC and IDC metastases further highlight differences between these two histologic BC subtypes. In ILC, *CDH1*, *PIK3CA*, *TBX3*, *FOXA1*, and *RUNX1* are the most commonly mutated genes [56]. Remarkably, genetic alterations in *PIK3CA*, *TBX3*, *FOXA1*, and *RUNX1* are associated with escape from oncogene-induced senescence and resistance to senescence [57,58,59,60,61] and systemic chemotherapy [62,63]. It is, therefore, tempting to speculate that the trend for ILC SLN metastases to contain fewer senescent cells compared to IDC SLN metastases (Table 6) may reflect the fact that ILC harbors more defects in genes that help cells to escape from senescence or to prevent oncogene-induced senescence.

In the context of aging, the extent to which the immune system is involved in regulating the number of senescent cells and whether age-related impairment of immune function contributes to the accumulation of senescent cells in the elderly is unknown [25]. Theoretically, the accumulation of senescent cells in breast tissue due to either aging or impaired immune surveillance could promote the development of breast cancer and later its progression. However, we did not observe an accumulation of senescent cells in the normal breast tissue surrounding tumors, even in patients at older ages (Table 4 and Figure 5). As we observed senescent tumor cells only within tumor tissue, we speculate that this reflects the ability of tumor cells to evade immune clearance. Recently, for example, it was shown that senescent cells foster the avoidance of immune recognition through MMPs in their SASP through a mechanism that involves MMP-dependent shedding of NKG2D-ligands that results in the suppression of NKG2D-mediated immunosurveillance [34]. Consistent with this notion, most breast carcinomas respond only inefficiently towards cancer immunotherapies, and a potential explanation might be that senescent breast cancer cannot be cleared by the immune system due to SASP-mediated suppression of immune surveillance. Thus, targeting senescent breast cancer cells with either senolytic or senomorphic drugs might render breast cancer more susceptible to the immune system and towards anti-cancer therapies.

In summary, this study demonstrates for the first time that senescent tumor cells exist within advanced human primary luminal breast carcinomas and that senescent tumor cells also exist within their matched lymph node metastasis. Several reports based on murine xenografts point to a metastasis-promoting function of senescent tumor cells. Since luminal breast carcinomas are characterized by late recurrence and poor susceptibility to chemotherapy and anti-tumor immunotherapies, it will be of importance to understand whether a correlation exists between senescence and tumor dormancy and whether senescence participates in tumor immune evasion. Further investigations focusing on the functional characterization of senescent breast cancer cells and their SASPs will be of importance to clarify the degree to which such senescent cells and their SASPs contribute to metastatic progression. Translationally, the targeting of senescent cancer cells or inhibiting the activity of SASP factors produced by senescent cancer cells holds promise as a strategy to improve breast cancer treatment and avoid late recurrence. It is of note that senolytic drugs such as ABT-263 induce apoptosis, leading to the clearance of therapy-induced senescent tumor cells [64]. Thus, it will be of particular interest to determine whether senolytic drugs can clear senescent breast cancer cells from breast carcinomas and thereby suppress therapy resistance, recurrence, and metastasis.

## 5. Conclusions

Oncogene-induced senescence is thought to constitute a barrier to carcinogenesis by arresting cells at risk of malignant transformation. Therefore, escape from senescence was regarded to be an absolute prerequisite for tumor initiation and progression. However, senescent tumor cells have been observed within several therapy-naïve human carcinomas, and numerous findings demonstrate that senescent cells promote tumor outgrowth and foster metastasis progression within in vivo models. Here, we investigated the degree to which senescent tumor cells exist within untreated human primary breast carcinomas and whether the presence of senescent cancer cells in primary breast tumors is recapitulated in their matched lymph node metastases. In patients with invasive luminal A and B breast carcinomas, we found broad similarities in the appearance of cancer cells between primary tumors and their corresponding metastasis. Analysis of lymph nodes from patients with other untreated breast cancer subtypes also revealed senescent tumor cells within metastatic lesions. Collectively, we show that senescent tumor cells exist within primary therapy-naïve breast carcinomas and metastatic lesions. These results suggest a potential role for senescent breast tumor cells during metastatic progression and support the notion that the targeting of senescent tumor cells or the SASP components they produce represents a novel avenue for improved treatment of breast and other cancers.

## Figures and Tables

**Table 1:** Relation of histological tumor type and histological grading of all patients.

Molecular Subtype		Grading		Histological Tumor Type	
Luminal	52	G1	5	Invasive ductal carcinoma	57
Luminal A	29				
Luminal B	23	G2	32	Invasive lobular carcinoma	6
HER2+	8				
ER+/HER2+	5	G3	21		
ER-/HER2+	3				
TNBC	3				

**Table 2:** Classification of the 63 sentinel lymph node metastases used in this study according to the St. Gallen molecular breast cancer samples.

Molecular Subtype	Luminal	Luminal A	Luminal B	HER2+	ER+/HER2+	ER−/HER2+	TNBC	Total Patients
Number of patients	**52**	29	23	**8**	5	3	**3**	63
Number of patients %	**82.5**	46	36.5	**12.7**	7.9	4.7	**4.8**	100

The number of patients with Luminal, HER2+ or triple negative breast cancer are highlighted in bold.

**Table 3:** SA-β-gal-positive tumor cells exist within primary breast carcinomas and in their matched lymph node metastases. Correlation between SA-β-gal staining and other senescence markers within primary luminal breast carcinomas and their matched SLN metastases.

Patients	Subtype	Histology	Grading	Sample	SA-β-gal	Ki67	p53	p16	p21
1	Luminal A	NST	1	Breast-Tumor	+	10%	low	low	-
				SLN	+	10%	high	-	-
2	Luminal A	NST	1	Breast-Tumor	+	5%	low	low	-
				SLN	+	N.T.	N.T.	N.T.	N.T.
3	Luminal A	NST	2	Breast-Tumor	+	15%	low	low	low
				SLN	+ +	30%	low	low	low
4	Luminal A	NST	2	Breast-Tumor	+	20%	low	low	-
				SLN	+	10%	low	-	-
5	Luminal A	NST	2	Breast-Tumor	+	20%	low	low	low
				SLN	+	20%	low	low	low
6	Luminal A	NST	2	Breast-Tumor	+	5%	low	-	low
				SLN	+	10%	low	-	low
7	Luminal A	NST	2	Breast-Tumor	−	<5%	-	-	-
				SLN	−	5%	low	low	-
8	Luminal A	NST	2	Breast-Tumor	−	10%	low	-	high
				SLN	+ +	10%	low	high	high
9	Luminal B	NST	3	Breast-Tumor	+	25%	high	high	-
				SLN	+	30%	high	high	-
10	Luminal B	NST	3	Breast-Tumor	+ +	30%	high	low	low
				SLN	+ +	30%	high	high	low

**Table 4:** SA-β-gal-positive tumor cells exist within primary breast carcinomas, but no SA-β-gal-positive epithelial cells exist in the normal breast tissue surrounding the tumor.

Patients	1	2	3	4	5	6	7	8	9	10
Age (years)	75	67	74	83	72	43	57	62	76	83
SA-β-gal positivity breast cancer tissue	+	+	+	+	+	+	−	−	+	++
SA-β-gal positivity adjacent breast tissue	−	−	−	−	−	−	−	−	−	−

SA-β-gal negative (−); SA-β-gal positive (+); SA-β-gal high positive (++).

**Table 5:** SA-β-gal-positive tumor cells exist within SLN metastases among all molecular breast cancer subtypes.

Molecular Subtype	Luminal	Luminal A	Luminal B	HER2+	ER+/HER2+	ER−/HER2+	TNBC	Total Patients
Number of patients	**52**	29	23	**8**	5	3	**3**	63
SA-β-gal high pos. (+ +)	**21 (40.3%)**	6 (20.6%)	15 (65.2%)	**7 (87.5%)**	4 (80%)	3 (100%)	**1 (33.3%)**	29 (46.0%)
SA-β-gal pos. (+)	**25 (48.0%)**	19 (65.5%)	6 (26.0%)	**1 (12.5%)**	1 (20%)	0 (0%)	**2 (66.6%)**	28 (44.0%)
SA-β-gal neg. (−)	**6 (11.5%)**	4 (13.7%)	2 (8.6%)	**0 (0%)**	0 (0%)	0 (0%)	**0 (0%)**	6 (9.5%)

The number of patients with Luminal, HER2+ or triple negative breast cancer are highlighted in bold.

**Table 6:** Differences in the appearance of SA-β-gal-positive tumor cells of SLN metastases exist between invasive ductal and invasive lobular carcinomas. (A) SA-β-gal high (+ +) and (+) positive tumor cells compared to SA-β-gal negative (−) tumor cells in IDC (NST) vs. ILC; (B) SA-β-gal high (+ +) positive tumor cells compared to SA-β-gal negative (−) tumor cells in IDC (NST) vs. ILC; (C) SA-β-gal (+) positive tumor cells compared to SA-β-gal negative (−) tumor cells in IDC (NST) vs. ILC.

Histology	SA-β-gal	Patients	A	B	C
invasive ductal (NST)	+ +	21	44 (95.7%)	21 (45.7%)	
	+	23			23 (50%)
	−	2	2 (4.3%)	2 (4.3%)	2 (4.3%)
invasive lobular	+ +	0	2 (33.3%)	0 (0%)	
	+	2			2 (33.3%)
	−	4	4 (66.7%)	4 (66.7%)	4 (66.6%)
Statistical significance			*p* < 0.0001	*p* < 0.0001	*p* < 0.0001

## Data Availability

The data presented in this study are available on request from C.L.C. and S.T.
